# Association between the minimal model of hip structure and risk of hip fracture in Chinese adults

**DOI:** 10.3389/fendo.2025.1558622

**Published:** 2025-03-18

**Authors:** Dan Zhao, Yawen Bo, Huiling Bai, Cuiping Zhao, Xinhua Ye

**Affiliations:** ^1^ Department of Endocrinology, The Second People’s Hospital of Changzhou, The Third Affiliated Hospital of Nanjing Medical University, Changzhou, Jiangsu, China; ^2^ Department of Geriatrics, The Second People’s Hospital of Changzhou, The Third Affiliated Hospital of Nanjing Medical University, Changzhou, Jiangsu, China

**Keywords:** hip structure, minimal model, delta, sigma, hip fracture

## Abstract

**Background:**

Multiple studies have indicated that the minimal model of hip structure can enhance hip fracture risk assessment. This study aimed to investigate the independent association between minimal model variables and hip fracture risk in Han Chinese individuals.

**Methods:**

This cross-sectional study included 937 Han Chinese patients (248 with hip fractures). Minimal model variables were calculated from the hip structural analysis, including bone mineral density (BMD), femoral neck width (FNW), and Delta and Sigma values.

**Results:**

This study included 937 patients (293 men; mean age = 68.3 years). In logistic regression analyses, BMD increase (per 0.1 g/cm^2^) correlated with a 45% reduction in the hip fracture risk (odds ratio [OR] = 0.55; 95% confidence interval [CI]: 0.45–0.68) after adjusting for all covariates. However, FNW (per 0.1 cm) and Sigma (per 0.01 cm) and Delta values (per 0.01 cm) were associated with increased risks (OR = 1.28; 95% CI: 1.18–1.37; OR = 1.06; 95% CI: 1.03–1.09; OR = 1.06; 95% CI: 1.03–1.09, respectively). When the Delta was >0.17 cm, the risk of hip fracture rose considerably by 13% (OR = 1.13; 95% CI: 1.08–1.18) for every 0.01 cm that the Delta value increased. The area under the curve (AUC) for hip fracture prediction from BMD alone was significantl lower than those of minimal model (0.781 vs 0.838, p <0.05).

**Conclusion:**

Large increases in FNW, Sigma and Delta values and notable declines in BMD were individually and significantly linked to a high hip fracture risk in Han Chinese adults. Our findings suggest that the minimal model of hip structure may improve hip fracture risk assessments.

## 1 Introduction

Hip fracture is a significant public health concern worldwide. The projected total annual incidence of hip fractures in many countries will nearly double between 2018 and 2050 (1). In 2019, the incidence and prevalence of hip fractures in China were approximately 2.0 million and 2.6 million, respectively, each representing approximately 1/9 of the global total cases (2). As China’s population continues to age in the forthcoming years, the country will encounter an increasing number of hip fracture-related issues.

Approximately 40 years after its inception, two-dimensional dual-energy X-ray absorptiometry (DXA), which measures the areal bone mineral density (aBMD) in the proximal femora, remains the most clinically used predictor of fracture risk (3, 4). However, academics are beginning to pay attention to the biomechanical implications of bone structural geometry on bone fragility (5–7). To capture the bone structure in cross sections at the femoral neck, Beck et al. created an eight-variable structural model known as hip structural analysis (HSA) using typical DXA imaging data (8). Rathbun et al. have reported that the proximal femur experiences a decline in bone structure and strength during hip fracture recovery that is significantly greater than that observed in older Caucasian men during normal aging (9). In native Chinese women, cortical thickness reduction or an increase in the buckling ratio may independently predict the risk of femoral neck fragility fractures, regardless of BMD (10).

However, the eight metrics typically documented using Beck’s HSA approach at each anatomical location were not autonomous (11). Utilizing HSA variables, Khoo et al. investigated the beam theory to develop a novel formulation, termed the minimum model (MM), which encompasses information equivalent to the eight structural geometric measures typically supplied at the femoral neck using the HSA technique. The MM consists of four parameters as follows: BMD and femoral neck width (FNW), along with two novel summary measures of internal bone distribution: Sigma and Delta (11). Prince et al. concluded that the clinical prediction of hip fractures was significantly enhanced by adding Delta measurements to hip BMD and age in elderly women (12). However, studies regarding the independent correlation between MM variables and the risk of hip fractures in Chinese adults are scarce.

This study aimed to investigate whether MM variables are significantly related to hip fracture risk in Han Chinese individuals, while controlling for all confounders. Furthermore, we implemented a receiver operating characteristic (ROC) analysis to compare the discriminative ability of MM against the use of femoral neck BMD alone.

## 2 Materials and methods

### 2.1 Study population

**Figure 1:**
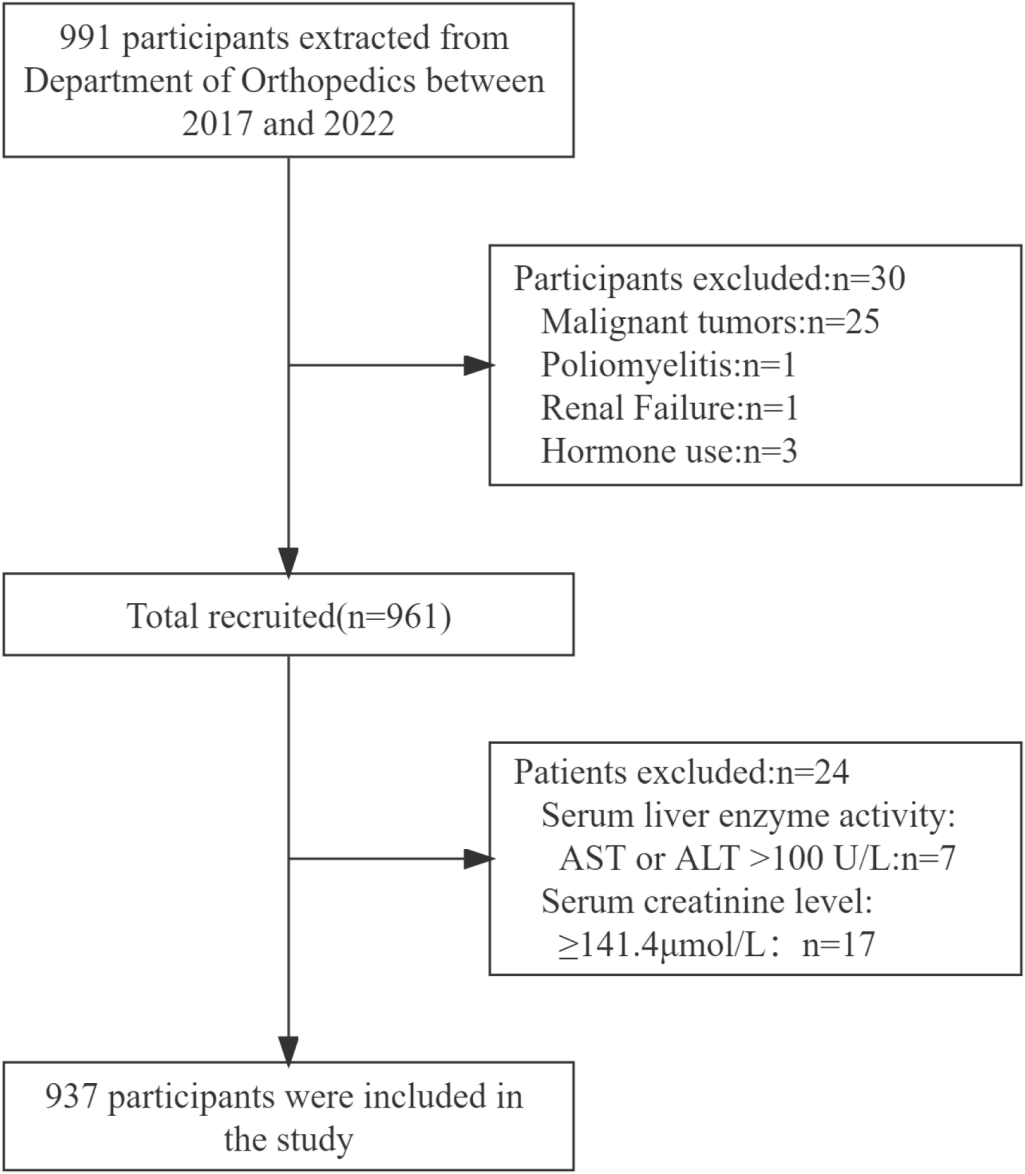
Flow diagram of the screening and enrollment processes of study participants. AST, aspartate aminotransferase; ALT, alanine aminotransferase.

This retrospective cross-sectional study was performed at the Department of Orthopedics of the Second People’s Hospital of Changzhou, Changzhou, Jiangsu, China. The inclusion and exclusion criteria for the participants were previously delineated (13). Participants with malignant tumors, poliomyelitis, renal failure, hormone use, elevated serum liver enzyme activity, or increased serum creatinine levels (n = 54) were excluded. Hip fractures were confirmed through a physician’s examination of the radiology reports, and the analysis included 937 participants, comprising 248 with hip fractures (**Figure 1**).

### 2.2 Measurements of clinical and laboratory parameters

Previous reports have documented the measurements of clinical and laboratory parameters (13). Weight (kg) and height (m) were assessed using a weighing scale, with the participants wearing light clothing and no shoes (RGZ-120-RT; Hengqi Inc., China). Body mass index (BMI; kg/m^2^)was calculated by dividing weight (kg) by height squared (m^2^). A glycosylated hemoglobin type-A1c (HbA1c) level > 6.5%, fasting glucose level > 7.0 mmol/L, and a self-reported history of a medical diagnosis of diabetes were considered indicators of diabetes.

Blood samples were collected from all participants within 24 h of admission following an overnight fast of at least 8 h. White blood cells (WBC), red blood cells (RBC), and platelets (PLT) were counted using a self-service hematology analyzer (XN-2800, Sysmex Inc.). Further biomarkers measured using a Siemens ADVIA-2400 included ALT, AST, alkaline phosphatase (ALP), albumin (ALB), blood urea nitrogen (BUN), creatinine (CCR), triglycerides (TG), high-density lipoprotein cholesterol (HDL-C), low-density lipoprotein cholesterol (LDL-C), fasting plasma glucose (FPG), and C-reactive protein (CRP). HbA1c levels were evaluated using the TOSOH G8-90SL. An ALIFAX TEST1-2730 device was used to assess the erythrocyte sedimentation rate (ESR).

### 2.3 BMD measurements

DXA scans of the hips were acquired using the Hologic Discovery Wi (Hologic Inc., Bedford, MA, USA). All scanners were operated by certified personnel. The bone density analyzed was defined as the projected aBMD of the left femoral neck in participants without fractures. The contralateral femoral neck was measured in patients with hip fractures.

The HSA program used in this study was created at Johns Hopkins University and incorporated into Hologic’s APEX product. As previously described (8), the HSA algorithm calculates structural parameters directly from the mass profiles: the total mineralized bone surface in the cross-section (CSA, cm^2^), cross-sectional moment of inertia (CSMI, cm^4^), section modulus (SM, cm^3^), and FNW (cm), the femoral neck area divided by the width of the neck box (the width of the femoral neck region was standardized at 1.5 cm).

### 2.4 Minimal model of hip structure

After revisiting the beam theory, the MM was conceived by KB and developed by Khoo et al. (11). The standard deviation of this mineral mass projection profile was Sigma (σ, cm), which is a measure of the variability of the mineral mass distribution along the mineral mass projection profile. Delta (δ, cm) represents the distance between the center of mass and the center of geometry for the mineral mass projection profile, indicating the section’s asymmetry. These two variables, in addition to BMD and FNW, constitute MM. Calculated from HSA measures, σ (Equation 1) and δ (Equation 2) can be defined as follows:


(1) $$\text{σ}=\sqrt{\frac{\text{CSMI}}{\text{CSA}}}$$

(2) $$\text{δ}=\left(\frac{\text{CSMI}}{\text{SM}}-\frac{\text{FNW}}{2}\right)$$

### 2.5 Statistical analysis

Patients were categorized into two groups according to the incidence of hip fractures: normally distributed continuous variables are expressed as means ± standard deviations (SDs), whereas skewed continuous variables are reported as medians with interquartile ranges. Categorical variables are presented as percentages (%). The chi-squared test, independent samples t-test, and Kruskal–Wallis test were used for categorical, normally distributed variables, and skewed distributions, respectively. For continuous variables with missing values of <2%, missing values were substituted with means or median values.

The independent association between MM variables and risk of hip fracture was assessed using multivariate logistic regression analysis. Both non-adjusted and multivariate-adjusted models were used, with the results presented as odds ratios (ORs) and 95% confidence intervals (CIs). These confounders were selected based on expert judgment, previous scientific literature, and all significant covariates identified in univariate analysis. Two models were developed; model I was adjusted for age, BMI, and gender, and model II was adjusted for model I + diabetes, WBC, RBC, PLT, ALT, ALP, ALB, CCR, TG, LDL-C, FPG, CRP, and ESR.

Interaction and stratified analyses were performed based on gender, the presence of diabetes mellitus, age, and BMI. To measure the subgroup heterogeneity, we multiplied the two predictor variables and added a new term to the model. We assessed the potential effect of the modification of diabetes and Sigma on hip fracture risk by calculating the interactions on both multiplicative and additive scales. Sigma was categorized into two groups (dichotomized). A cross-product interaction term was incorporated into the logistic regression model to evaluate the multiplicative interactions. The additive interaction was evaluated using two indices: the relative excess risk due to the interaction (RERI) and the attributable proportion due to the interaction (AP) (14). Both the RERI and AP were 0 if there was no additive interaction.

Generalized additive models (GAM) were used to discern nonlinear relationships, considering that the MM variables were continuous, and potential confounders were adjusted for. Utilizing smoothed curves, a two-segment linear regression model was developed to ascertain the threshold effects. The threshold levels of Delta were established through a recursive methodology that included identifying turning points in conjunction with predefined intervals as well as selecting turning points that produced a maximum likelihood model. A log-likelihood ratio test was used to evaluate the two-segment linear regression model against a nonlinear linear model.

The evaluation of each model’s predictive capability was conducted through receiver operating characteristic (ROC) curve analysis. The area under the ROC curve (AUC) served as a metric to assess the risk of hip fractures. The AUC of minimal model of hip structure was compared to the AUC of femoral neck BMD alone.

The R statistical software (version 4.2.2, http://www.R-project.org, The R Foundation) and Free Statistics Analysis Platform (version 1.9, Beijing, China, http://www.clinicalscientists.cn/freestatistics) were used to conduct all analyses (15). Free Statistics is a software program that provides user-friendly interfaces for common analysis and data visualization. The software uses R as the core statistical engine with a graphical user interface created in Python. Statistical significance was defined as a two-sided p < 0.05.

## 3 Results

### 3.1 Baseline characteristics of the study participants

This study included 937 Han Chinese individuals (293 men and 644 women). The baseline clinical and biochemical features of the patients stratified according to the incidence of hip fractures are presented in **Table 1**. The age of the participants ranged from 31 to 99 years, with a mean age of 68.3 years (SD = 10.5). Participants with fractures exhibited older age and greater height, FNW, and Sigma and Delta values than those without fractures. Additionally, they demonstrated significantly lower CSA, SM, BMI, and BMD values.

**Table 1:** Baseline characteristics of participants.

Variables	Total (n = 937)	Without fracture (n = 689)	With fracture (n = 248)	*P*-value
gender, %				0.804
Male	293 (31.3)	217 (31.5)	76 (30.6)	
Female	644 (68.7)	472 (68.5)	172 (69.4)	
Age, years	68.3 ± 10.5	66.6 ± 9.5	73.0 ± 11.8	< 0.001
Diabetes, %				0.755
No	732 (78.1)	540 (78.4)	192 (77.4)	
Yes	205 (21.9)	149 (21.6)	56 (22.6)	
Weight, kg	62.9 ± 11.1	64.5 ± 11.0	58.5 ± 10.4	< 0.001
Height, m	1.58 ± 0.08	1.57 ± 0.08	1.60 ± 0.08	< 0.001
BMI, kg/m^2^	25.1 ± 3.9	26.0 ± 3.7	22.7 ± 3.5	< 0.001
WBC, 10^9^/L	6.2 (5.1, 7.8)	5.8 (4.9, 6.9)	8.0 (6.6, 9.8)	< 0.001
RBC, 10^9^/L	4.3 ± 0.5	4.4 ± 0.5	4.1 ± 0.5	< 0.001
PLT, 10^9^/L	211.9 ± 64.8	216.8 ± 59.1	198.3 ± 77.1	< 0.001
ALT, U/L	16.0 (12.0, 22.9)	17.0 (12.8, 23.7)	14.0 (11.0, 19.1)	< 0.001
AST, U/L	21.0 ± 8.6	20.9 ± 8.6	21.1 ± 8.7	0.684
ALP, U/L	80.6 ± 26.7	79.7 ± 26.3	83.3 ± 27.9	0.066
ALB, g/L	42.5 ± 4.2	43.5 ± 4.0	39.8 ± 3.8	< 0.001
BUN, mmol/L	5.8 (4.8, 6.9)	5.8 (4.8, 6.9)	5.7 (4.7, 7.0)	0.581
CCR, μmol/L	62.6 ± 16.8	62.0 ± 16.2	64.4 ± 18.4	0.05
TG, mmol/L	1.3 (0.9, 1.9)	1.4 (1.0, 2.0)	1.0 (0.8, 1.4)	< 0.001
HDL-C, mmol/L	1.4 ± 0.3	1.4 ± 0.3	1.4 ± 0.3	0.994
LDL-C, mmol/L	2.6 ± 0.8	2.7 ± 0.7	2.4 ± 0.8	< 0.001
FPG, mmol/L	6.0 ± 1.8	5.9 ± 1.5	6.5 ± 2.5	< 0.001
CRP, mg/L	5.0 (3.5, 13.6)	5.0 (2.6, 6.1)	31.0 (9.7, 65.3)	< 0.001
HbA1c, %	6.2 ± 1.1	6.2 ± 1.0	6.2 ± 1.3	0.545
ESR, mm/h	21.0 (11.0, 34.0)	19.0 (9.0, 29.0)	28.0 (16.2, 46.0)	< 0.001
CSA, cm^2^	2.572 ± 0.580	2.659 ± 0.548	2.331 ± 0.598	< 0.001
CSMI, cm^4^	2.372 ± 0.895	2.380 ± 0.835	2.347 ± 1.047	0.616
SM, cm^3^	1.201 ± 0.380	1.236 ± 0.356	1.105 ± 0.426	< 0.001
BMD, g/cm^2^	0.785 ± 0.167	0.827 ± 0.153	0.668 ± 0.149	< 0.001
FNW, cm	3.458 ± 0.358	3.384 ± 0.317	3.664 ± 0.385	< 0.001
Sigma, cm	0.947 ± 0.096	0.934 ± 0.089	0.984 ± 0.103	< 0.001
Delta, cm	0.225 ± 0.081	0.211 ± 0.069	0.264 ± 0.098	< 0.001

Data presented are mean ± SD, median (Q1–Q3), or N (%).

BMI, body mass index; WBC, white blood cell; RBC, red blood cell; PLT, platelet; ALT, alanine aminotransferase; AST, aspartate aminotransferase; ALP, alkaline phosphatase; ALB, albumin; BUN, blood urea nitrogen; CCR, creatinine; TG, triglycerides; HDL-C, high-density lipoprotein cholesterol; LDL-C, low-density lipoprotein cholesterol; FPG, fasting plasma glucose; HbA1c, glycosylated hemoglobin type-A1c; CRP, C-reactive protein; ESR, erythrocyte sedimentation rate; BMD, bone mineral density; FNW, femoral neck width.

### 3.2 Logistic regression analyses

The risk of hip fracture increased with higher FNW (OR = 1.26; 95% CI: 1.20–1.32), Sigma values (OR = 1.06; 95% CI: 1.04–1.07), and Delta values (OR = 1.09; 95% CI:1.07–1.11) in the univariate logistic regression analyses. Additionally, a negative correlation was identified between the risk of hip fracture and BMD (OR = 0.47; 95% CI: 0.42–0.54). Age, BMI, WBC, RBC, PLT, ALT, ALP, ALB, CCR, TG, LDL-C, FPG, and CRP levels, and ESR were correlated with the risk of hip fracture, as adjusted in model II. Other factors such as AST, BUN, HDL-C, and HbA1c levels did not show significant associations (**Supplementary Table 1**).

The results of the multivariate logistic regression analysis are presented in **Table 2**. The association remained significant after controlling for age, BMI, and gender. In model II, BMD increase (per 0.1 g/cm^2^) was associated with a 45% decrease in the risk of hip fracture (OR = 0.55; 95% CI: 0.45–0.68); however, FNW (per 0.1 cm), Sigma (per 0.01 cm), and Delta (per 0.01 cm) measurements were associated with an increased risk of hip fracture (OR = 1.28; 95% CI: 1.18–1.37; OR = 1.06; 95% CI: 1.03–1.09; OR = 1.06; 95% CI: 1.03–1.09).

**Table 2:** Association between simplified hip structure analysis method and the risk of hip fracture.

Variable	Nonadjusted	*P*-value	Adjust I	*P*-value	Adjust II	*P*-value
BMD, per 0.1 g/cm^2^	0.47 (0.42~0.54)	<0.001	0.52 (0.44~0.60)	<0.001	0.55 (0.45~0.68)	<0.001
FNW, per 0.1 cm	1.26 (1.20~1.32)	<0.001	1.32 (1.24~1.40)	<0.001	1.28 (1.18~1.37)	<0.001
Sigma, per 0.01 cm	1.06 (1.04~1.07)	<0.001	1.07 (1.05~1.09)	<0.001	1.06 (1.03~1.09)	<0.001
Delta, per 0.01 cm	1.09 (1.07~1.11)	<0.001	1.08 (1.05~1.10)	<0.001	1.06 (1.03~1.09)	<0.001

Data are presented as ORs and 95% CIs.

Adjusted model I was adjusted for age, body mass index, and gender; adjusted model II was adjusted for model I + diabetes, white blood cells, red blood cells, platelets, alanine aminotransferase, alkaline phosphatase, albumin, creatinine, triglycerides, low-density lipoprotein cholesterol, fasting plasma glucose, C-reactive protein, and erythrocyte sedimentation rate.

BMD, bone mineral density; FNW, femoral neck width.

### 3.3 Subgroup analyses

**Figure 2:**
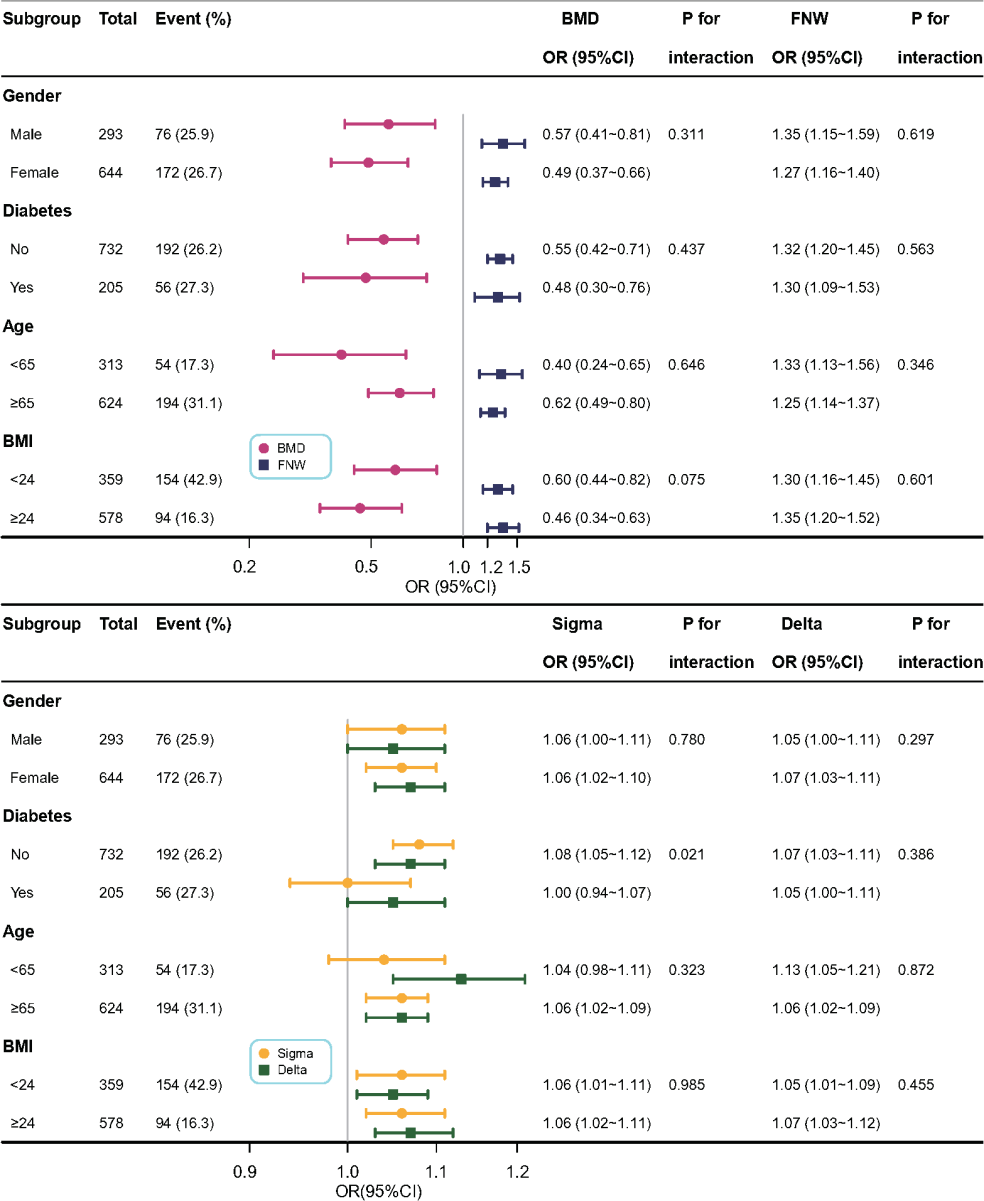
Association between BMD, FNW, Sigma, Delta, and the risk of hip fracture in Subgroup analyses based on gender, diabetes, age, and BMI. Each stratification adjusted for all factors (age, BMI, gender, diabetes, white blood cells, red blood cells, platelets, alanine aminotransferase, alkaline phosphatase, albumin, creatinine, triglycerides, low-density lipoprotein cholesterol, fasting plasma glucose, C-reactive protein, erythrocyte sedimentation rate) except the stratification factor itself. BMI, body mass index; BMD, bone mineral density; FNW, femoral neck width.

Subgroup analyses were conducted to further investigate the impact of age, gender, BMI, and diabetes on study outcomes. The results of these analyses are shown in **Figure 2**. The effect sizes of BMD, FNW, and Delta on the risk of hip fractures remained robust and reliable. Nevertheless, the association between Sigma and the risk of hip fracture was not statistically significant in the patients aged <65 years (OR = 1.04; 95% CI: 0.98–1.11) and diabetes (OR = 1.0; 95% CI: 0.94–1.07) groups. No interactions were detected, except for the impact of diabetes and Sigma on the risk of hip fracture (p for multiplicative interaction < 0.05). Subsequently, we analyzed additive interaction and observed no interactions between diabetes and Sigma regarding the risk of hip fractures (all p > 0.05; **Supplementary Table 2**).

### 3.4 GAM

**Figure 3:**
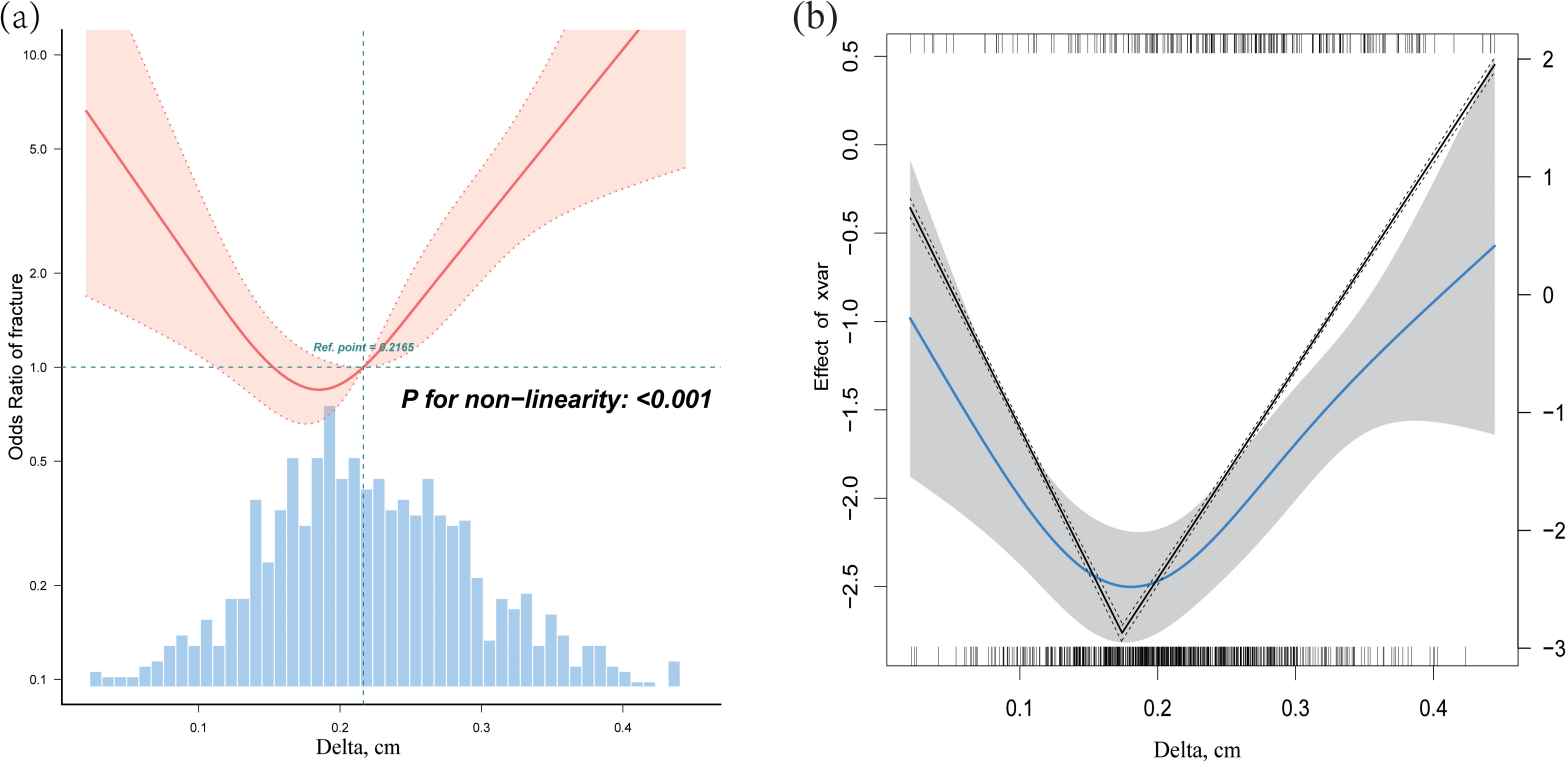
**(a)** A curved relationship between Delta and the risk of hip fracture. Adjustment factors included age, body mass index, gender, diabetes, white blood cells, red blood cells, platelets, alanine aminotransferase, alkaline phosphatase, albumin, creatinine, triglycerides, low-density lipoprotein cholesterol, fasting plasma glucose, C-reactive protein, and erythrocyte sedimentation rate. **(b)** Threshold levels of Delta were determined using a recursive approach that involved selecting turning points along with predefined intervals and selecting turning points that yielded a maximum likelihood model.

A multivariate logistic regression model based on restricted cubic splines was used to fit the data with confounders adjusted in accordance with model II. The estimated dose–response curve revealed a substantial linear association between BMD, FNW, Sigma, and the risk of hip fracture (**Supplementary Figure 1**; p for nonlinearity > 0.05). A curved rather than a linear relationship was observed between the Delta measurement and risk of hip fracture after adjusting for all covariates. Using a two-segment linear regression model, the Delta value was 0.17 cm (**Figure 3**).

### 3.5 Threshold effect analysis

Above the threshold, the risk of hip fracture was significantly increased by 13% (OR = 1.13; 95% CI: 1.08–1.18) for every 0.01 cm Delta increase. When Delta was <0.17 cm, a decrease in Delta was linked to a higher risk of hip fracture; however, this association was not statistically significant (p > 0.05) (**Table 3**).

**Table 3:** Threshold effect analysis of the association between Delta values and the risk of hip fracture.

Outcome:	OR (95% CI)	*P*-value
One-line linear regression model	1.06 (1.03~1.09)	<0.001
Two-piecewise linear regression model		
< 0.17cm	0.89 (0.76~1.04)	0.151
≥ 0.17cm	1.13 (1.08~1.18)	<0.001
Log-likelihood ratio test		<0.001

Delta per change 0.01 cm. ORs were adjusted for age, body mass index, gender, diabetes, white blood cells, red blood cells, platelets, alanine aminotransferase, alkaline phosphatase, albumin, creatinine, triglycerides, low-density lipoprotein cholesterol, fasting plasma glucose, C-reactive protein, and erythrocyte sedimentation rate.

### 3.6 ROC analysis

**Figure 4:**
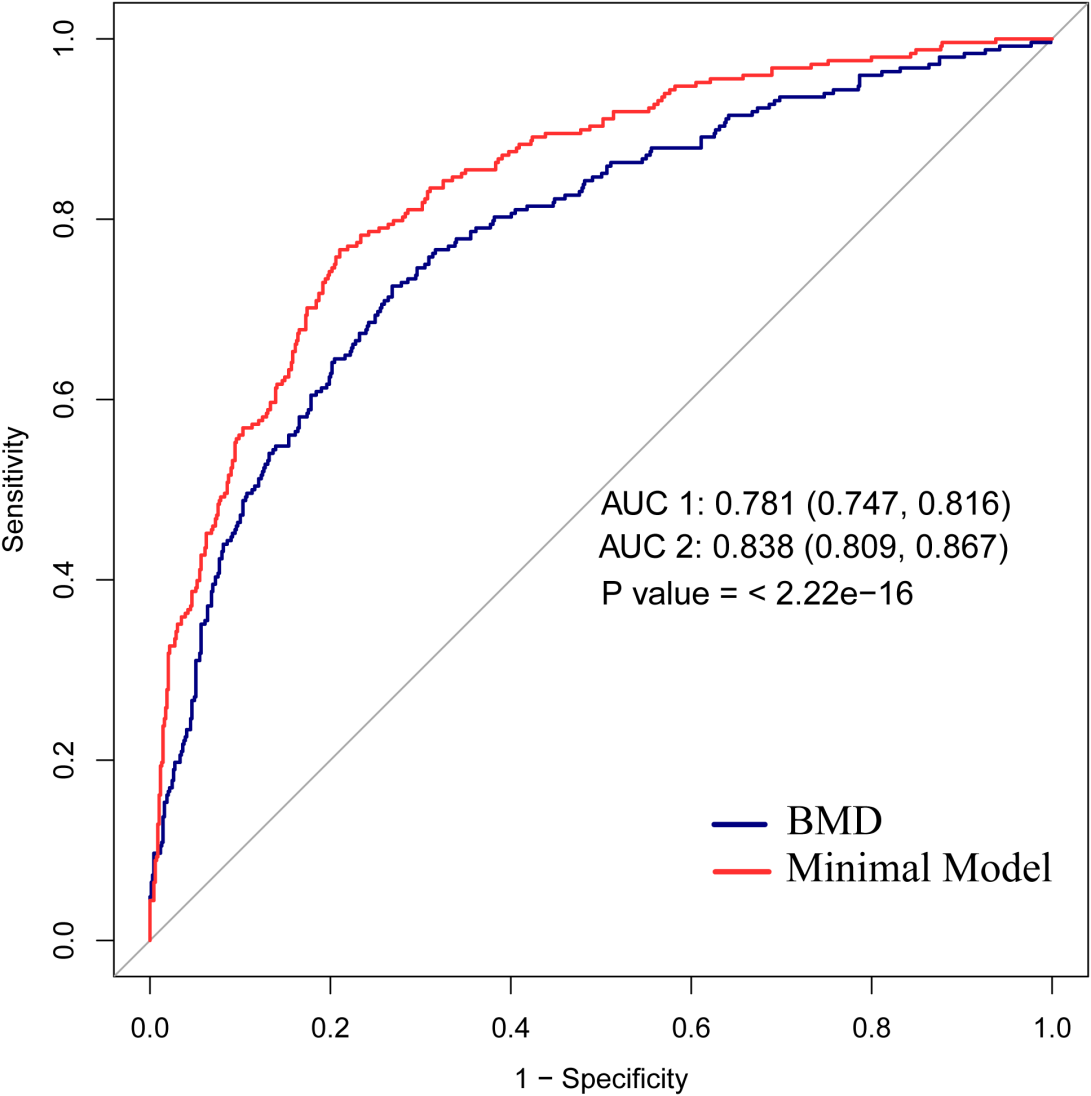
ROC analysis and AUC for hip fracture prediction. AUC1: BMD; AUC2: Minimal Model. BMD, FNW, Delta, and Sigma values comprised the minimal model variables. C statistics for the difference between AUC1 (0.781) and AUC2 (0.838), P < 0.001. BMD, bone mineral density; FNW, femoral neck width.

**Figure 4** presents the C-statistic for sensitivity and specificity regarding hip fracture risks. The area under the curve for predicting hip fractures based on BMD was 0.781 (0.747, 0.816). The AUC2 for minimal model of hip structure was significantly greater than that of BMD (0.838 vs 0.781, p < 0.05).

## 4 Discussion

This retrospective cross-sectional study included 937 Han Chinese adults, of whom 248 had hip fractures. Large increases in FNW and Sigma and Delta values and notable declines in BMD were separately and significantly linked to a higher risk of hip fracture after adjusting for age, BMI, gender, and clinical risk factors. The subgroup and additive interaction analyses confirmed the robustness of these associations. Apart from Delta measurement, we observed a linear association between BMD, FNW, Sigma, and the risk of hip fracture in the GAM analysis.

An increased Delta value indicates a downward shift in the center of mass, suggesting a decrease in bone mass in the upper region of the femoral neck cross-section (16). This deficiency in the superior segment is commonly acknowledged to contribute to hip fractures by facilitating buckling, a type of compressive failure (17). Prince has reported that each SD increment of Delta corresponded to a hazard ratio (HR) of 1.51 for the risk of femoral neck fracture (95% CI: 1.17–1.94) (12), aligning with findings reported by Khoo et al. (18, 19). In our study, an increase in Delta (per 0.01 cm) was linked to a 6% higher risk of hip fracture (OR = 1.06; 95% CI: 1.03–1.09).

Interestingly, the Delta value and risk of hip fracture showed a curved link. A decrease in Delta value was associated with a lower hip fracture risk (OR = 0.89; 95% CI: 0.76–1.04) when the Delta value decreased to <0.17 cm. Above the threshold, the risk of hip fracture increased considerably by 13% (OR = 1.13; 95% CI: 1.08–1.18) for every 0.01 cm increase in the Delta value. To the best of our knowledge, this study is the first to comprehensively elucidate the dose–response relationship, providing new insights into the prediction and treatment of femoral neck fractures.

However, the relationship between Sigma and fracture risk remains unclear. In a study involving elderly women from Beijing, Khoo et al. indicated a low Sigma (per SD) as a risk factor (OR = 0.70; 95% CI: 0.54–0.92) (19). However, Prince did not identify a significant association in the Perth Longitudinal Study of Aging in Women Sigma [(per SD); HR = 0.72; 95% CI: 0.46–1.10] (12). In Khoo’s study, each SD increment of Sigma corresponded to a 116% increase in the risk of hip fracture among Chinese men (OR = 2.16; 95% CI: 1.24–3.78) (18). Our data indicate a significant association between Sigma and the risk of hip fractures [Sigma (per 0.01 cm); OR = 1.06; 95% CI: 1.03–1.09], applicable to both genders. Greater Sigma values reflect a reduction in trabecular bone mass near the center of mass of the femoral neck cross-section because they indicate a larger mineral mass distribution (17).

In this study, we observed a lower BMD, greater FNW, wider mineral mass distribution around the center of mass, and an inferomedial shift in the center of mass, which were significantly associated with a higher risk of hip fractures, which is consistent with previous research findings (20–22). Structural inadequacy of the femoral neck may be correlated with the prevalent remodeling imbalance associated with aging (7, 23, 24). The limited mechanical requirements of middle and old age may be accommodated by the retention of the inferomedial femoral neck cortex and preferential loss of the superolateral cortex (25). This alteration may confer a protective effect during physiological stance loading as demonstrated by Fox et al. Conversely, it should reduce strength during bending during falls (26).

Multiple studies indicate that low BMD is the most sensitive predictor of hip fractures among clinical risk factors (27, 28). However, the present study demonstrated that minimal model of hip structure had a much greater prediction ability for hip fractures compared to BMD alone (AUC: 0.838 vs 0.781, p < 0.05). Our findings indicate that minimal model of hip structure may improve hip fracture risk assessments.

Our study has several limitations. First, this study was conducted on Han Chinese individuals; therefore, the findings may not apply to other ethnic groups. Second, the cross-sectional retrospective design prevented us from confirming a causal relationship between hip MM variables and risk of hip fractures. Third, we cannot rule out the possibility that unmeasured confusing elements could be responsible for the observed correlations, even after adjusting for confounding factors to the fullest extent possible. Finally, the two groups revealed some differences in baseline characteristics, and the participants with hip fractures were older than those without hip fractures. Nevertheless, we managed the most pertinent variables in the logistic regression models. Consequently, multicenter randomized controlled trials with robust designs are essential to validate our findings.

## 5 Conclusions

Large increases in FNW, Sigma and Delta values, and notable declines in BMD were separately and significantly linked to a high risk of hip fractures in Han Chinese adults. The Delta value and risk of hip fracture showed a curved link. Thus, our findings suggest that the minimal model of hip structure may improve hip fracture risk assessments.

## Data Availability

The datasets presented in this article are not readily available because the data contains sensitive patient information that is subject to strict confidentiality and ethical guidelines. The data includes personal health records and identifiable information, which cannot be shared publicly to protect patient privacy and comply with data protection regulations. Requests to access the datasets should be directed to Dan Zhao, gleam2024@163.com.
