# Supplementary material for: Association between the minimal model of hip structure and risk of hip fracture in Chinese adults
Source: Front Endocrinol (Lausanne). 2025 Mar 18;16:1558622. doi: 10.3389/fendo.2025.1558622 (PMC11958231; doi:10.3389/fendo.2025.1558622)
Supplement: Supplementary file 1 [file DataSheet1.docx]

**Association between the minimal model of hip structure and risk of hip fracture in Chinese adults**

**Frontiers in Endocrinology**

DanZhao^1†^, Yawen BO^1†^, Huiling Bai^1^, Cuiping Zhao^2*†^, Xinhua Ye^1*†^

^1^ Department of Endocrinology, The Second People's Hospital of Changzhou, the Third Affiliated Hospital of Nanjing Medical University, Changzhou, Jiangsu, China

**^2^** Department of Geriatrics, The Second People's Hospital of Changzhou, the Third Affiliated Hospital of Nanjing Medical University, Changzhou, Jiangsu, China

^*^Corresponding authors:

Xinhua Ye, MA, Department of Endocrinology,

The Second People's Hospital of Changzhou,

No.68 Gehu Road, Changzhou, Jiangsu, China

E-mail: [czyxh2000@163.com](mailto:czyxh2000@163.com)


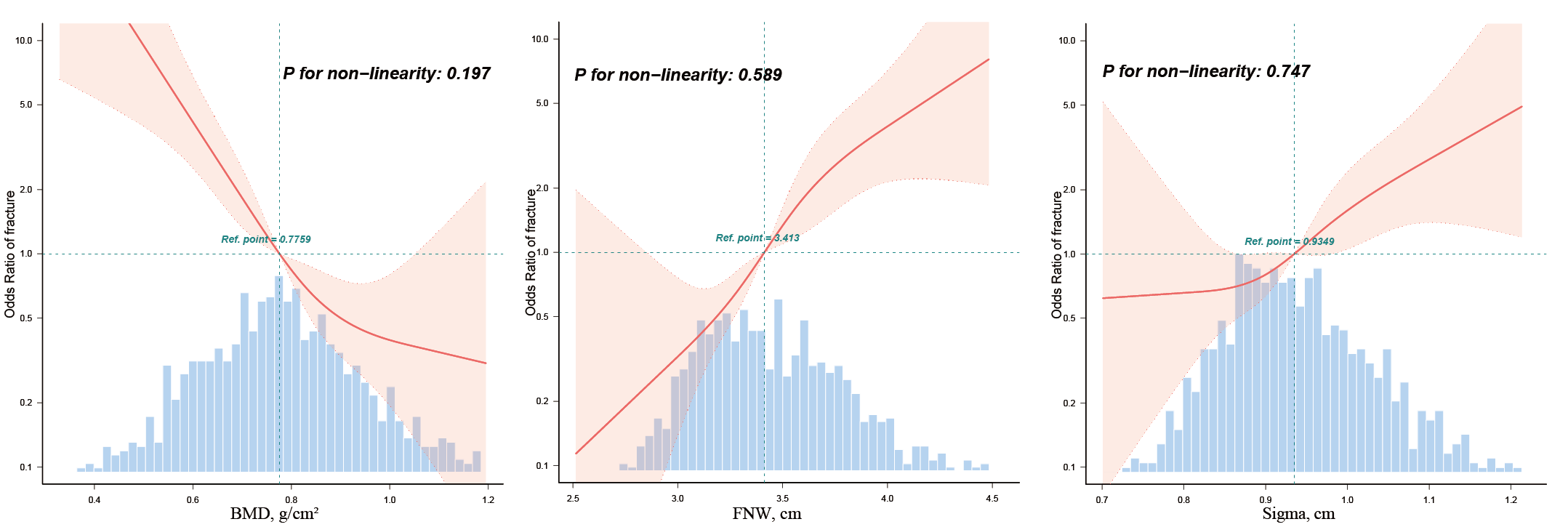


**Supplementary Figure 1** A linear association between bone mineral density, femoral neck width, Sigma and the risk of hip fracture. Adjustment factors included age, body mass index, gender, diabetes, white blood cells, red blood cells, platelets, alanine aminotransferase, alkaline phosphatase, albumin, creatinine, triglycerides, low-density lipoprotein cholesterol, fasting plasma glucose, C-reactive protein, and erythrocyte sedimentation rate.

Abbreviations: BMD, bone mineral density; FNW, femoral neck width.

**Supplementary Table 1** Univariate analysis of the risk of hip fracture

| Variable | OR_95CI | *P*-value |
| --- | --- | --- |
| Age, years | 1.07 (1.05~1.08) | <0.001 |
| Weight, kg | 0.95 (0.93~0.96) | <0.001 |
| Height, per 0.1m | 1.65 (1.37~1.99) | <0.001 |
| BMI, kg/m^2^ | 0.77 (0.73~0.81) | <0.001 |
| WBC, 10^9^/L | 1.45 (1.35~1.57) | <0.001 |
| RBC, 10^9^/L | 0.30 (0.22~0.41) | <0.001 |
| PLT, 10^9^/L | 1.00 (0.99~1.00) | <0.001 |
| ALT, U/L | 0.97 (0.96~0.99) | 0.001 |
| AST, U/L | 1.00 (0.99~1.02) | 0.684 |
| ALP, U/L | 1.00 (1.00~1.01) | 0.069 |
| ALB, g/L | 0.77 (0.74~0.81) | <0.001 |
| BUN, mmol/L | 1.00 (0.95~1.04) | 0.874 |
| CCR, μmol/L | 1.01 (1.00~1.02) | 0.05 |
| TG, mmol/L | 0.35 (0.26~0.46) | <0.001 |
| HDL-C, mmol/L | 1.00 (0.65~1.54) | 0.994 |
| LDL-C, mmol/L | 0.50 (0.41~0.62) | <0.001 |
| FPG, mmol/L | 1.17 (1.08~1.26) | <0.001 |
| CRP, mg/L | 1.07 (1.06~1.09) | <0.001 |
| HbA1c, % | 1.04 (0.91~1.18) | 0.545 |
| ESR, mm/h | 1.02 (1.02~1.03) | <0.001 |
| BMD, per 0.1 g/cm^2^ | 0.47 (0.42~0.54) | <0.001 |
| FNW, per 0.1 cm | 1.26 (1.20~1.32) | <0.001 |
| Sigma, per 0.01 cm | 1.06 (1.04~1.07) | <0.001 |
| Delta, per 0.01 cm | 1.09 (1.07~1.11) | <0.001 |

Note: data are presented as ORs and 95% CIs

Abbreviations: BMI, body mass index; WBC, white blood cell; RBC, red blood cell; PLT, platelet; ALT, alanine aminotransferase; AST, aspartate aminotransferase; ALP, alkaline phosphatase; ALB, albumin; BUN, blood urea nitrogen; CCR, creatinine; TG, triglycerides; HDL-C, high-density lipoprotein cholesterol; LDL-C, low-density lipoprotein cholesterol; FPG, fasting plasma glucose; HbA1c, glycosylated hemoglobin type-A1c; CRP, C-reactive protein; ESR, erythrocyte sedimentation rate; BMD, bone mineral density; FNW, femoral neck width.

**Supplementary Table 2** Multiplicative and additive interactions between diabetes and Sigma on the risk of hip fracture

| Variable |  | Variable |  | Multiplicative  interaction | Additive  interaction | |
| --- | --- | --- | --- | --- | --- | --- |
|  |  |  |  |  |  |  |
|  |  |  | OR (95% CI) | OR (95% CI) | RERI (95% CI) | AP (95% CI) |
| Diabetes | No | Low Sigma | 1 | 0.31(0.10, 0.94)^*^ | -1.87(-3.60, -0.15) | -2.3(-5.38, 0.07) |
|  |  | High Sigma | 2.71(1.55, 4.72)^*^ |  |  |  |
|  | Yes | Low Sigma | 0.98(0.40, 2.43) |  |  |  |
|  |  | High Sigma | 0.82(0.29, 2.26) |  |  |  |

^*^p < 0.05

Adjustment factors included age, body mass index, gender, white blood cells, red blood cells, platelets, alanine aminotransferase, alkaline phosphatase, albumin, creatinine, triglycerides, low-density lipoprotein cholesterol, fasting plasma glucose, C-reactive protein, and erythrocyte sedimentation rate.
